# Feasibility study for marker‐based VMAT plan optimization toward tumor tracking

**DOI:** 10.1002/acm2.12892

**Published:** 2020-06-11

**Authors:** Azeez A. Omotayo, Sankar Venkataraman, Niranjan Venugopal, Boyd McCurdy

**Affiliations:** ^1^ Division of Medical Physics CancerCare Manitoba Winnipeg MB Canada; ^2^ Department of Physics and Astronomy University of Manitoba Winnipeg MB Canada; ^3^ Department of Radiology University of Manitoba Winnipeg MB Canada

**Keywords:** constraints, fiducial marker, plan optimization, tumor tracking, VMAT

## Abstract

This work investigates the incorporation of fiducial marker‐based visibility parameters into the optimization of volumetric modulated arc therapy (VMAT) plans. We propose that via this incorporation, one may produce treatment plans that aid real‐time tumor tracking approaches employing exit imaging of the therapeutic beam (e.g., via EPID), in addition to satisfying purely dosimetric requirements. We investigated the feasibility of this approach for a thorax and prostate site using optimization software (*MonArc*). For a thorax phantom and a lung patient, three fiducial markers were inserted around the tumor and VMAT plans were created with two partial arcs and prescription dose of 48 Gy (4 fractions). For a prostate patient with three markers in the prostate organ, a VMAT plan was created with two partial arcs and prescription dose 72.8 Gy (28 fractions). We modified *MonArc* to include marker‐based visibility constraints (“hard”and “soft”). A hard constraint (HC) imposes full visibility for all markers, while a soft constraint (SC) penalizes visibility for specific markers in the beams‐eye‐view. Dose distributions from constrained plans (HC and SC) were compared to the reference nonconstrained (NC) plan using metrics including conformity index (CI), homogeneity index (HI), gradient measure (GM), and dose to 95% of planning target volume (PTV) and organs at risk (OARs). The NC plan produced the best target conformity and the least doses to the OARs for the entire dataset, followed by the SC and HC plans. Using SC plans provided acceptable dosimetric tolerances for both the target and OARs. However, OAR doses may be increased or decreased based on the constrained marker location and number of trackable markers. In conclusion, we demonstrate that visibility constraints can be incorporated into the optimization together with dosimetric objectives to produce treatment plans satisfying both objectives. This approach should ensure greater clinical success when applying real‐time tracking algorithms, using VMAT delivery.

## 1 Introduction

Radiotherapy is commonly used for effective management of many cancers. However, tumors in regions such as lungs, liver, pancreas, and breast move due to respiration. This introduces additional positioning uncertainty for thoracic and abdominal tumors. These tumors may move up to 30 mm during treatments^1^ due to breathing. The 2006 AAPM Task Group report on respiratory management^2^ recommends that for tumor motion exceeding 5 mm, motion management techniques should be used. However, more recent work recommends using motion management for all procedures.^3^ Motion management may be beneficial to account for nonrespiratory motion encountered in some disease sites, such as transient rectal gas motion observed in prostate treatment.

The tumor volume may be significantly displaced during the treatment due to respiration or other involuntary motion. For regular tumor motion caused by respiration, a treatment margin around the tumor may be added to ensure full dose coverage of the gross tumor volume (GTV) during its movement throughout the patient’s respiratory cycle. However, the increased margin also exposes a larger volume of the surrounding healthy normal tissues around the target to the treatment dose, making it more difficult to deliver sufficient dose to the tumor without damaging the healthy tissue. As a result, methods and treatment techniques to reduce the treatment margin and corresponding exposure of normal tissues to treatment dose are necessary. Motion management proposals implemented to date include real‐time tumor tracking using the machine’s multileaf collimator (MLC) jaws or patient couch,^4^, ^5^, ^6^ respiratory gating,^7^, ^8^ breath control, etc. For stereotactic body radiation therapy (SBRT), where smaller treatment field sizes and very high doses (up to 20 Gy per fraction in 3–5 fractions) are used to treat tumors, it is even more crucial to minimize the dose to the surrounding healthy tissues.^9^

Tumor motion tracking methods during radiotherapy are desirable as they can potentially improve treatment accuracy and target dose conformity, while further sparing organs at risk (OARs) and surrounding healthy tissues. Recently proposed tumor motion compensation can be divided mainly into three techniques: breath control; respiratory gating, and dynamic tumor tracking.^2^ Breath control and gating techniques, however, require the therapeutic beam application only during a specified breath‐hold level.^1^ Dynamic tumor tracking mainly involves two processes: (a) localizing the target in real‐time and (b) simultaneously steering or collimating the therapeutic beam to adapt to the target motion. Thus, tumor tracking allows continuous and dynamic treatment dose delivery to the tumor without forced or controlled breathing, leading to better patient comfort and reduced treatment duration. However, it is important to note that while real‐time (i.e., dynamic) tumor tracking is an active research topic in radiation oncology, it is not yet available commercially for conventional C‐arm linear accelerators.

In clinical practice, radio‐opaque fiducial markers implanted in or near to the tumor have been used for image‐guided radiation therapy (IGRT) and also for real‐time tumor target localization for several tumor sites, via projection imaging. Approaches typically use unmodulated, open fields due to the risk of shielding the fiducial markers when using full volumetric modulated arc therapy (VMAT) delivery methods. Li *et al.* ^10^ utilized a Bayesian approach for real‐time three‐dimensional (3D) tumor localization combining the 2D projections from kV x‐ray imager available on linear accelerators during treatment, with the aid of gold cylindrical markers, using motion trajectories of a lung and pancreas patient reproduced in a phantom. They reported 3D localization errors of 1.5 and 0.8 mm for the lung and pancreas,^10^ respectively. However, the maximum *a priori* method employed in their Bayesian approach depends on appropriate hyperparameter selection for optimal results, which needs to be chosen before treatment (e.g., via x‐ray images acquired during patient setup). Also, irregularities in tumor motion patterns may require incorporation of a dynamic motion model.

Azcona *et al.* ^11^ implemented an automated fiducial marker detection algorithm for prostate VMAT using 2D MV cine EPID images. Their approach was based on a combined criteria using template matching and image intensity information, using a dynamic search area that predicts the fiducial position and updates in real‐time to track the marker and to account for marker occlusion by MLC leaves. However, the 3D fiducial position is predicted if its expected position lies in open beam which is derived from planning CT. While promising, the approach suffers from sparse image data from few projections due to small arc of gantry rotation,^11^ especially where all fiducial markers are blocked. Another limitation of this approach is effect of false positives, as a threshold correlation metric value between the EPID and planning CT positions were used. The work acknowledges that it is challenging to apply this approach for tracking where there is fast fiducial marker motion or occlusion of all the fiducials by MLC leaves. In summary, the prior literature indicates that the more reliably fiducials are visible, the more robust and effective the real‐time tracking algorithm will be. This observation motivates the current work.

The expected visibility of the fiducial markers in the beam's eye view (BEV) overlaid by the MLC apertures at each gantry angle (i.e., control point) is available in the planning process; we can use this information in the plan optimization strategy. We propose that by incorporating marker visibility in the plan optimization step, one may produce a treatment plan that ensures a higher chance of successful application of real‐time tracking techniques, in addition to satisfying dosimetric requirements. Very few studies have investigated the incorporation of markers into the plan optimization.^12^ Ma *et al.* ^12^ investigated the feasibility of four‐dimensional (4D) IMRT planning with the inclusion of a fiducial marker constraint into the optimization, for one pancreas and lung cancer patient. They introduced a time‐resolved, synchronized MLC segments to the 10 breathing phase of the 4DCT and added a fiducial visibility constraint to the simulated annealing probability, whereby a fixed penalty term is used such that segmented fields are rejected or accepted if some or all of the markers are blocked during the simulated annealing process. They reported only a slight degradation in the target dose distribution occurred when all markers were forced to be seen (i.e., a “hard” constraint) in the segmented fields. Similar results were deduced for the OARs. However, they did not report results for VMAT and explored only one penalty term in the optimization.

Our study is the first to incorporate marker‐based visibility constraints into VMAT plan optimization. In this work, we use radiotherapy‐based optimization development software (*MonArc*) to optimize VMAT‐based plans, using a combination of dosimetric and fiducial marker visibility constraints. We demonstrate the feasibility of this approach by introducing visibility constraints into the optimization in addition to the standard dosimetric objectives, and produce treatment plans that are clinically acceptable in terms of the predefined dosimetric tolerances. We hypothesize that our approach will produce acceptable treatment plans that should facilitate real‐time tumor tracking, solely based on the marker visibility parameter, which can be successfully implemented in the clinic. The next step of investigating the effect on a real‐time tumor tracking method on the created plans will be the subject of future work.

## 2 Methods and Materials

### 2.A Data acquisition and treatment plan optimization

An averaged 4DCT dataset of 10 phases of the breathing cycle was used to create a VMAT‐based plan with two partial arcs in a Varian Eclipse Treatment Planning System (TPS) (version 13.6.23). The target structures (i.e., planning target volume = PTV; gross target volume = GTV; and clinical planning volume = CTV) and OAR structures were all contoured in the Eclipse TPS. The VMAT plans were then exported to an inverse planning and optimization research software, *MonArc*.

*MonArc* utilizes an optimization approach and framework, termed progressive resolution optimization (PRO), proposed by Otto in his seminal VMAT paper.^13^ This approach to VMAT optimization was commercialized as RapidArc^®^ by Varian Medical Systems in 2008. The original version of *MonArc* was provided to our group for research purposes by Dr. Otto. The optimization algorithm in *MonArc* utilizes an objective function that finds the optimal shapes and weights of all the MLC segments/apertures in a VMAT delivery, based on the prescribed dose and specified dose constraints to targets and OARs provided by the user. Details of the *MonArc* optimization process can be found in Ref. [13] and related publications.^14^, ^15^

*MonArc* was modified to incorporate the visibility of the markers in the BEV of each MLC segment associated with individual control points in the plan, by adding visibility constraints to the objective function. VMAT plans developed with different marker‐based visibility constraints were investigated as described below in Section 2.C. Dose distributions from plans with added fiducial marker‐based visibility constraints were then compared to VMAT plans with no such constraints (i.e., only dosimetric constraints), to analyze the discrepancies. All *MonArc* optimized plans were imported into Eclipse and doses recalculated with either Acuros XB (Acuros External Beam) or AAA (Anisotropic Analytical Algorithm) algorithms, to take advantage of the dose visibility and comparison tools available in the commercial Eclipse TPS.

### 2.B Phantom and patient data selection

**Fig. 1:**
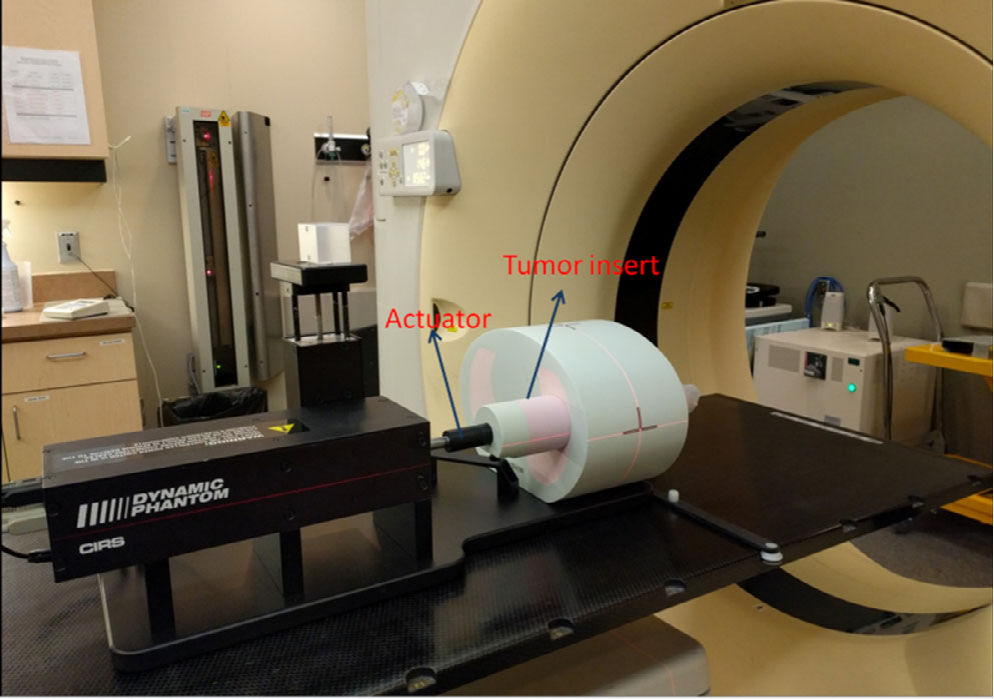
CIRS Dynamic Thorax phantom used for 3D tumor motion simulation.

In order to evaluate the feasibility of our approach, a thorax phantom was used for validation. Lung tumor 3D motion was simulated using a dynamic thorax phantom (CIRS Inc., Norfolk, VA, USA). The phantom consists of a motorized rod with tumor inserts of different sizes made of tissue equivalent materials (Fig. 1). Different preset motion waveforms are available and also patient‐specific breathing waveforms that have been previously recorded can be used to reproduce the motion. We used a 1‐cm diameter tumor insert for this study, with three artificial fiducial markers implanted around the tumor (within the PTV) in order to emulate BEV visibility during real‐time tracking. The phantom also had embedded OAR structures including vertebrae and spinal cord.

The thorax phantom patient had PTV and ITV target structures (9.0 and 1.7 cc, respectively) and standard OAR structures including spinal cord (1.2 cc), vertebrae (38.8 cc), and lungs (1954 cc). The prescription dose for the target was 48 Gy over four fractions using a 6 MV flattening filter‐free x‐ray (6X‐FFF) beam from a Varian TrueBeam^TM^ linear accelerator, with two partial arcs (Arc 1: 315°–179.9° clockwise; Arc 2: 179.9°−315° counterclockwise) and no collimator rotation.

In addition to the thorax phantom, the feasibility of the technique was also demonstrated on an example clinical lung and prostate VMAT patient dataset which also each had three implanted fiducial markers. We chose a prostate patient data because it possesses less homogeneity in structures and also because fiducial markers are readily used in our clinic at the tumor site. However, any patient dataset with markers in or around the PTV can be used.

The lung SBRT VMAT patient had PTV and GTV target structures (27 and 8 cc, respectively) and standard OAR structures including esophagus (24 cc), heart (615 cc), left lung (2110 cc), spinal cord (46 cc), and ribs (27 cc). The prescription dose to the PTV was 48 Gy over four fractions with two partial arcs (Arc 1: 330°–179° clockwise; Arc 2: 179°−330° counterclockwise) and collimator rotation (Arc 1: 30°; Arc 2: 330°), using a 6 MV flattening filter‐free x‐ray beam (6X‐FFF) from a Varian TrueBeam^TM^ linear accelerator. All *MonArc* optimized plans were re‐imported and dose calculated in TPS using the Acuros XB (version 13.6.23) algorithm.

The prostate VMAT patient had PTV and CTV target structures (107 and 39 cc, respectively) and standard OAR structures including rectum (100 cc), bladder (125 cc), head‐of‐femur (175 cc), and penile bulb (1.1 cc). The prescription dose to the PTV was 72.8 Gy over 28 fractions with two partial arcs (Arc 1: 181°‒179° clockwise; Arc 2: 179°‒181° counterclockwise) and collimator rotation (Arc 1: 30°; Arc 2: 330°), using a 6 MV x‐ray beam (6X) from a Varian Clinac^TM^ linear accelerator. Similarly, all *MonArc* optimized plans were re‐imported into Eclipse TPS and dose calculated using the AAA (version 13.6.23) algorithm.

### 2.C Optimization algorithm (with marker visibility constraints)

The VMAT optimization algorithm in *MonArc* follows the PRO approach, which uses a variation of the direct aperture optimization (DAO) technique.^16^ At the beginning of the optimization, the gantry arc rotation is coarsely sampled using only a few static beam angles (e.g., 4–13 beams) with aperture or segments that conform to the projection of the targets in the BEV. Thence, more beam samples are added between preexisting angles until a fine angular resolution is achieved that approximates the full gantry rotation. As opposed to using a probability distribution based on a preset system temperature used in simulated annealing, the changes in the beam weights and MLC leaf positions are randomly sampled from a uniform distribution.^17^ Thus, MLC leaves and beam weights are used as optimization parameters.

A new beam sample is inserted midway between two existing beams and its initial MLC leaf positions are linearly interpolated from the MLC positions of the two adjacent beams.^13^ The weight of the new sample is based on a redistribution of weights from its immediate neighbors. Beam samples are successively added to the set, until a desired sampling frequency is reached. In order to reach a favorable solution, the optimization is guided by an objective function based on dose–volume constraints as detailed by Bortfeld *et al.* ^18^ Multiple minimum and maximum dose–volume constraints can be defined for each target, OAR or surrounding tissue structure. During optimization, MLC leaf position and beam weight changes are only accepted if the cost function decreases. The cost of each constraint is determined via a quadratic dose‐difference function multiplied by a preset priority value (i.e., user‐controlled). The total plan cost equals the sum of the individual constraint costs.^13^, ^17^ Their approach has been shown to produce high quality, deliverable VMAT treatment plans.

The individual dose objectives for a plan lead the optimization toward the intended dose distribution for the target and critical organs. To explore the effect of the inclusion of visibility objectives, fiducial marker visibility requirements were programmed as optimization parameters, and were implemented as either “hard” or “soft” constraints. The projection points of the fiducial markers can be seen in the BEV such that they can be processed as structures and included in the optimization. During the optimization, as the aperture shapes and dose weights are progressively added, the inclusion of new MLC apertures are either accepted or rejected based on the desired marker visibility constraint (“hard” or “soft”). That is, a particular marker‐based constrained plan requires 100% visibility of some marker structure in the BEV. Only after this realization is the dose‐computed and new apertures added to continue the iteration.

A hard constraint (HC) imposes complete and full visibility for all implanted markers (thereby avoiding any shielding or blockage of the markers by the MLC segmented fields/apertures) in the BEV, while a soft constraint (SC) restricts visibility for a specific set of markers (thereby penalizing MLC blockage) in the BEV. Thus, for the scenarios considered here with three markers implanted, there are six possible combinations of SC plans (i.e., SC12, SC13, SC23, SC1, SC2, and SC3 whereby 1, 2, and 3 represents the marker index such that SC1 = soft‐constrained plan for marker 1; SC12 = soft‐constrained plan for markers 1 and 2, etc.) and just one HC (i.e., HC ≡ SC123 = soft‐constrained plan for all available three markers). Hence, there are seven constrained plan combinations in total and all were investigated along with the nonconstrained (NC) plans for the phantom, lung, and prostate dataset. NC plans are plans (either in Eclipse TPS or *MonArc*) whereby no marker visibility parameter is included during the optimization. Table 1 details the plan and optimization constraints used on the dynamic thorax phantom, while Tables 2 and 3 details the constraints used on the lung and prostate patients, respectively.

**Table 1:** Dose constraints for VMAT plan optimization in the dynamic thorax phantom

Dose constraints			
Targets: PTV; ITV			
Structures	Min dose volume/dose	Max dose volume/dose	Prescribed dose
PTV	100%/48Gy	10%/52Gy	48Gy
ITV/GTV	100%/44Gy	10%/48Gy	44Gy
Relevant OARs: spinal cord, vertebral body, and lungs			
OAR (spinal cord)	50% volume receiving ≤ 10 Gy		
OAR (vertebral body)			
OAR (lungs)			

PTV, planning target volume; ITV, internal target volume; OARs, organs at risk; VMAT, volumetric modulated arc therapy.

**Table 2:** Dose constraints for VMAT plan optimization in the lung SBRT patient.

Dose constraints				
Targets: PTV; GTV				
*Structures*		Min dose volume/ Dose	Max dose volume/dose	Prescribed dose
PTV		100%/48Gy	0%/52Gy	48Gy
ITV/GTV		100%/48Gy	0%/52Gy	48Gy
Relevant OARs: lung (Left), esophagus, heart, and ribs Specific plan constraints				
OAR (lung)	30% volume receiving <13 Gy (Left Lung) 30% volume receiving <10 Gy (Right Lung)			
OAR (esophagus)	20% volume receiving <20 Gy 0.2% volume receiving ≤30 Gy			
OAR (heart)	3% volume receiving <25 Gy			
OAR (ribs)	4% volume receiving <32 Gy			

PTV, planning target volume; OARs, organs at risk; GTV, gross target volume; VMAT, volumetric modulated arc therapy; SBRT, stereotactic body radiation therapy.

**Table 3:** Dose constraints for VMAT plan optimization in the prostate patient.

Dose constraints				
Targets: PTV; CTV				
Structures		Min dose volume/ dose	Max dose volume/dose	Prescribed dose
PTV		100%/72.8Gy	0%/75Gy	72.8Gy
CTV		100%/72.8Gy	0%/75Gy	72.8Gy
Relevant OARs: bladder, rectum, and penile bulb Specific plan constraints				
OAR (bladder)	25% volume receiving ≤ 65Gy 50% volume receiving ≤ 47Gy			
OAR (rectum)	10% volume receiving ≤ 68Gy 25% volume receiving ≤ 55Gy 40% volume receiving ≤ 42Gy			
OAR (penile bulb)	Mean dose: < 50Gy			

PTV, planning target volume; CTV, clinical target volume; OARs, organs at risk; VMAT, volumetric modulated arc therapy.

### 2.D Marker visibility and trackability

We hypothesize that including the marker visibility constraints into the optimization would aid real‐time tumor tracking, especially for dynamic MLC (DMLC) tracking. Most marker‐based tracking algorithms in the literature do require the marker to be seen in a chosen template of a region of interest. However, we did not implement real‐time tracking methods in this work. Instead, a quantitative metric for marker visibility was defined and evaluated. For our purposes, we will use the number of markers fully visualized at each gantry angle or control point of the optimized VMAT plans as a metric to define visibility and “trackability”; trackability is a measure of the visibility for specific markers — which are either inside or in close proximity to the PTV — in the BEV. Hence, our chosen quantitative metric will be assumed to depict trackability. As expected, a HC plan will elicit 100% trackability as all markers should be visualized in the BEV by the fields of the MLC apertures.

### 2.E Plan comparison metrics

We compare plan dosimetry qualitatively using cumulative dose–volume histogram (DVH) plots as well as quantitatively using dose metrics including mean dose, maximum dose, conformity index (CI), homogeneity index (HI), and gradient measure (GM). The target dose CI represents how closely the prescription dose conforms to the target volume and is calculated as the ratio of volume enclosed by the prescribed isodose surface divided by the target volume, that is,

$$\mathrm{Conformity}\;\mathrm{index}\left(\mathrm{CI}\right)=\frac{\mathrm{reference}\;\mathrm{isodose}\;\mathrm{volume}}{\mathrm{target}\;\mathrm{volume}}$$

The CI describes the conformity and the overlap of the prescription isodose with respect to the PTV. Thus, an ideal CI would be a value of 1, although typically the CI is greater than 1. Note that the “reference isodose volume” is the volume that receives 95% of the prescribed (Rx) dose, which is dependent on the plan normalization method selected in this study (i.e., 100% of Rx dose delivered to 95% target volume).

The dose GM^19^ represents the dose slope measured between the equivalent sphere radius of the prescription (Rx) isodose and half‐prescription isodose.

$Gradient\;measure\;\left(\mathrm{GM}\right)=\frac{\left(Rx-50\%\;Rx\;dose\right)}{average\;distance\;between\;Rx\;isodose\;and\;50\%\;Rx\;isodose}$

Thus, a smaller GM indicates a higher dose gradient around the target structure volume. A large GM translates into a large dose spillage outside the target. Both CI and GM are reported in the Eclipse TPS only for the selected target volume (i.e., PTV).

The HI is defined by the Radiation Therapy Oncology Group (RTOG 1993) as:

$$\mathrm{Homogeneity}\;\mathrm{index}\left(\mathrm{HI}\right)=\frac{\mathrm{maximium}\;\mathrm{target}\;\mathrm{isodose}\;\mathrm{volume}}{\mathrm{reference}\;\mathrm{isodose}\;\mathrm{volume}}$$

The ideal HI value is 1, and HI values greater than 1 signify a less homogeneous plan. Note that the “maximum target isodose volume” is the maximum dose to the PTV, which is always more than 100% of the Rx dose, as well as the “reference isodose volume” as defined above. Hence, as expected HI will always be greater than 1 in our study.

To analyze and compare plans for the patient datasets, we also reported an average index of the above quantitative metrics and used it along with our internal physician‐created clinical dose metrics for hypofractionated prostate and SBRT lung VMAT. Metrics used for the phantom and lung VMAT plans:

1. PTV: dose to 95% volume greater than 95% Rx (D95 > 95%);
2. OAR lung: dose delivered to 30% volume less than 13Gy (D30 < 13Gy);
3. OAR esophagus: volume receiving 18.8Gy less than 5 cm^3^ (V18.8 < 5cc);
4. OAR heart: volume receiving 28Gy less than 15 cm^3^ (V28 < 15cc);

For the prostate VMAT patient plans, metrics used include:

1. PTV: dose delivered to 99% of the target volume (D99);
2. OAR rectum: volume receiving 74Gy is less than 3 cm^3^ (V74 < 3cc);
3. OAR bladder: volume receiving 65Gy is less than 25% of Rx dose (V65 < 25%);
4. OAR penile bulb: mean dose not to exceed 50Gy.

## 3 Results

### 3.A Marker‐based constrained optimization on lung phantom

**Fig. 2:**
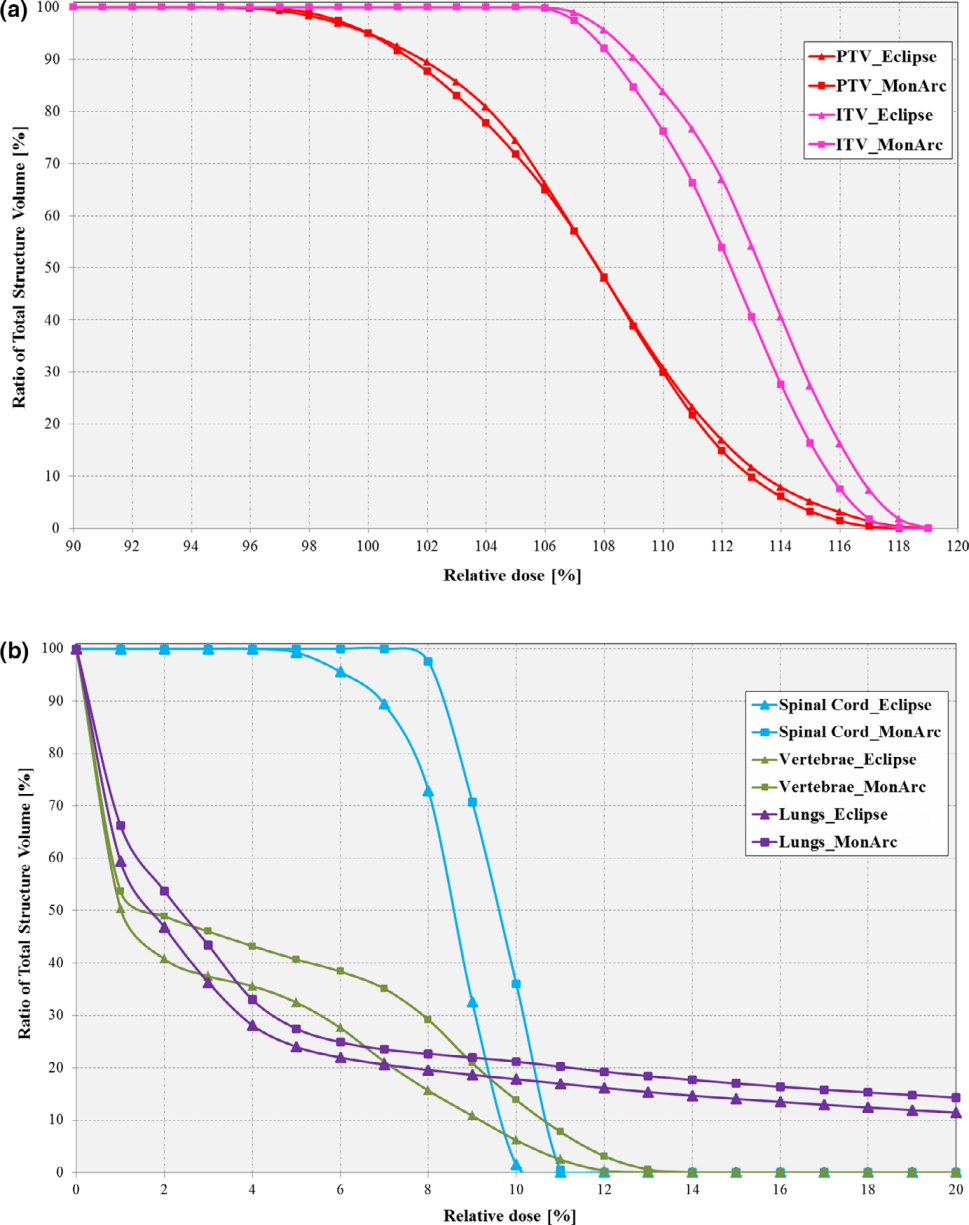
Zoomed‐in dose–volume histogram comparison between nonconstrained clinical Eclipse‐based (triangle ) and *MonArc*‐based (square ) volumetric modulated arc therapy plans for (a) target structures (ITV [pink]; PTV [red]) and (b) organs at risks (Spinal cord; Vertebrae; Lungs) on the dynamic thorax phantom.

Figures 2(a)–2(b) shows the DVH and dose distribution comparison between the NC clinical VMAT plan in Eclipse^®^ TPS and the *MonArc*‐based plans for the thorax phantom. As can be observed, the *MonArc*‐based plan is inferior in terms of dose conformity to the targets [Fig. 2(a)]. This is because it uses the research version of the multi‐resolution PRO which has been updated and modified in its Eclipse^®^ implementation. However, both plans were able to deliver 100% of prescription dose to 95% of the PTV. Similar results were obtained for the spinal cord and vertebrae OARs [Fig. 2(b)]. This shows that the *MonArc*‐based optimization produced acceptable dosimetric results relative to the clinically used Eclipse^®^ TPS. Henceforth, all further optimizations reported use *MonArc*.

**Fig. 3:**
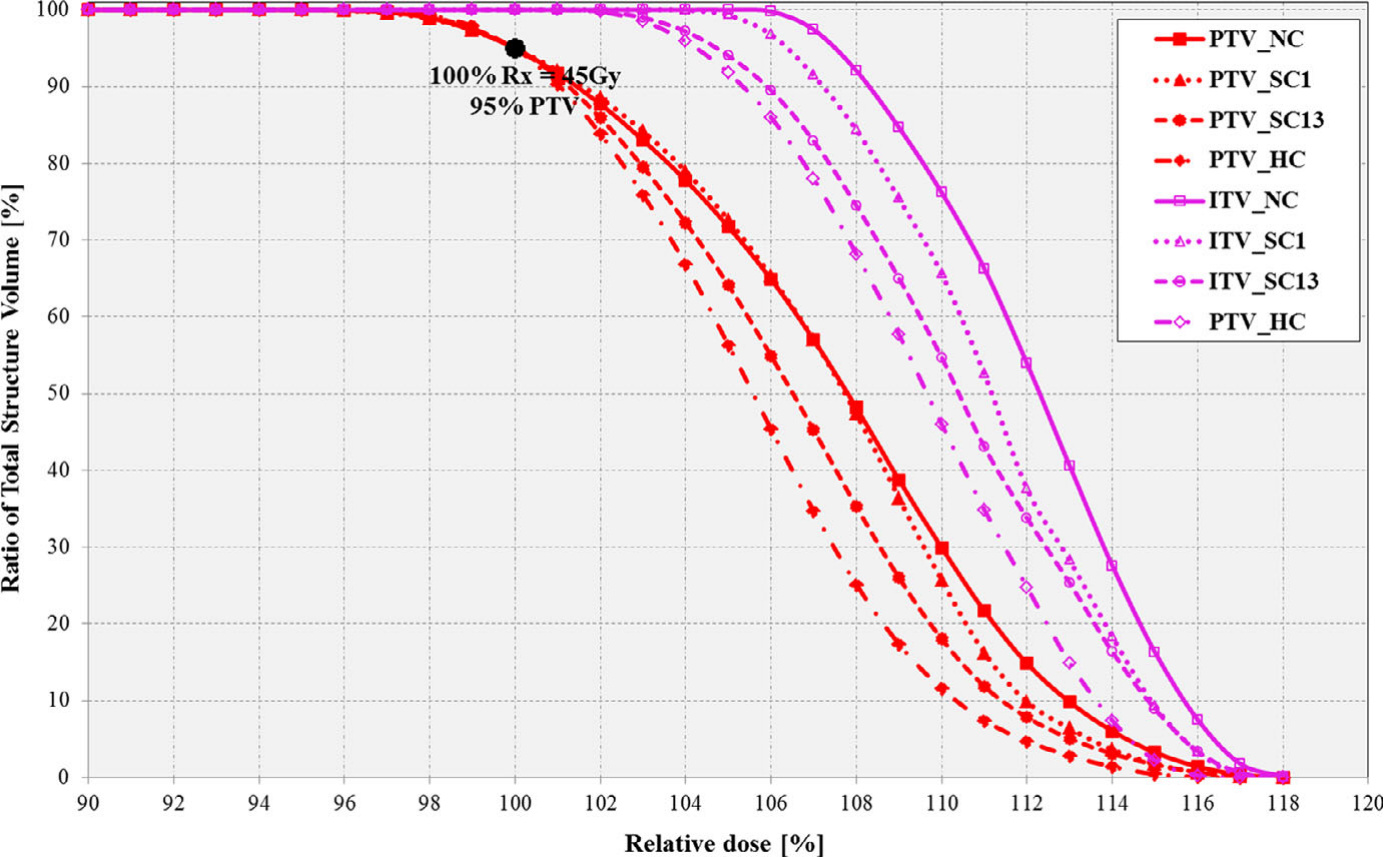
Dose–volume histogram (zoomed‐in) for target structures (PTV and ITV) using *MonArc*‐based non‐, soft‐ and hard‐constrained optimized plans in the dynamic thorax phantom.

**Fig. 4:**
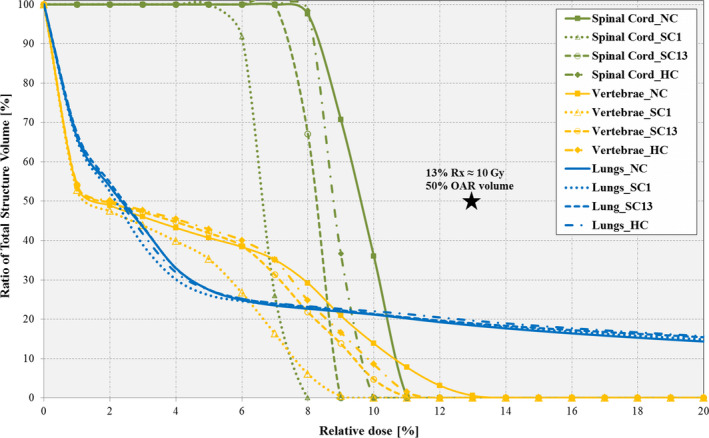
Dose–volume histogram (zoomed‐in) for organs at risk (OARs) (Spinal Cord [green]; Vertebrae [yellow]; Lungs [blue]) using *MonArc*‐based non‐, soft‐, and hard‐constrained optimized plans in the dynamic thorax phantom. Highlights (black star) represent labeled specific dose‐quality threshold for all three OARs.

The markers are visible as required in the MLC aperture field openings at each sample gantry control point (not shown here). All investigated plans show similar target dose distribution and coverage, with slightly differing OAR coverage. Figures 3 and 4 shows the targets’ and OAR’s zoomed‐in DVH plots for all the plans, respectively. The HC plan offered the best optimal dose coverage for the PTV target, as opposed to the NC plan which provided the worst target dose–volume coverage. The SC13 plan (i.e., soft‐constrained plan for two specific markers 1 and 3) provided the next best optimal target dose–volume coverage, while the SC1 plan (i.e., SC plan for one specific marker 1) provided the least optimal coverage (Table 7). Similar results were obtained for the lung, spinal cord and vertebrae OARs with no notable significant differences in the mean doses among all the constrained plans. However, for the spinal cord it can be observed that the SC1 and SC2 plans had the lowest dose, while the NC plan had the highest cord dose.

### 3.B Marker‐based constrained optimization on lung patient

**Fig. 5:**
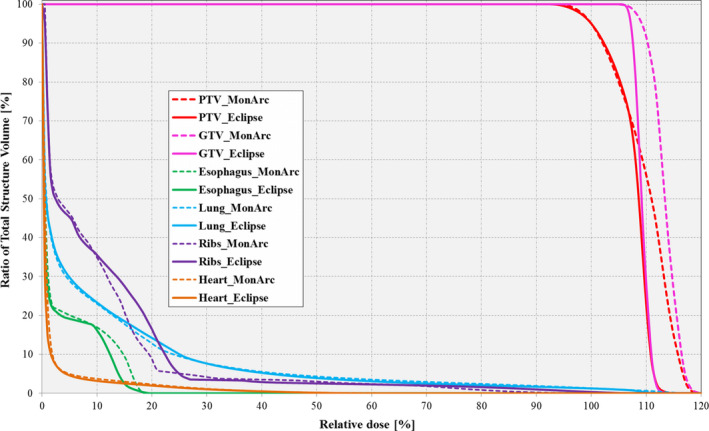
Dose–volume histogram comparison between Eclipse‐based and *MonArc*‐based volumetric modulated arc therapy plans for the target structures (GTV [pink]; PTV [red]) and organs at risks (Esophagus [green]; Lung [blue]; Ribs [purple] and Heart [orange]) on the sample lung SBRT patient. Both plans are nonconstrained on the markers.

Figure 5 shows the DVH comparison between the NC clinical Eclipse‐ and *MonArc*‐based lung SBRT VMAT patient plans. As can be seen, the *MonArc*‐based plan is slightly inferior in terms of dose conformity to the target. However, both plans satisfied the internal site‐specific clinical dose tolerances for the targets and OARs.

**Fig. 6:**
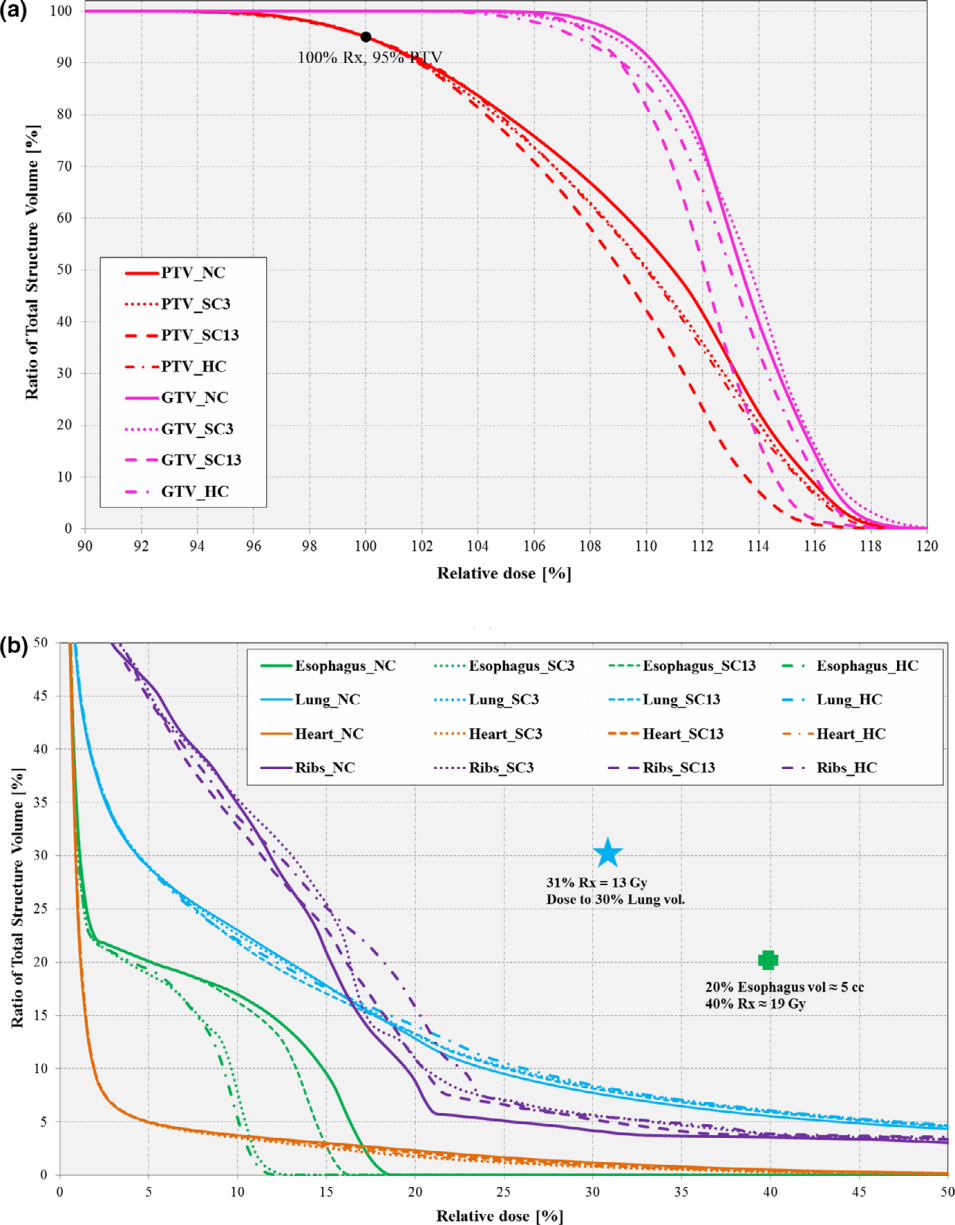
Dose–volume histogram comparison for (a) targets (GTV [pink] and PTV [red]) and (b) organs at risk (OARs) (Esophagus [green]; Lung [blue] and Heart [orange]) between *MonArc*‐based nonconstrained (NC), soft‐constrained (SC), and hard‐constrained (HC) volumetric modulated arc therapy plans on lung SBRT patient. Highlights (circle, cross, star) represent labeled target and OAR‐specific dose‐quality thresholds.

Figures 6(a)–6(b) shows the targets and OAR’s zoomed‐in DVH plots, respectively. As observed, the SC13 (i.e., Inferior + Superior marker) plan offered the best optimal dose coverage for the PTV and GTV, followed by the HC plan. However, it should be emphasized that there was no significant difference in the PTV coverage, as all the plans produced dosimetrically acceptable coverage and conformity for the PTV target as defined by internal clinical standards. For the lungs [see Fig. 6(b) and Table 8], all constrained plans met the clinical dose threshold D30% <13 Gy (i.e., dose to 30% lung volume is <13 Gy), including the NC plan. Also for the esophagus OAR, all constrained plans met the clinical dose threshold V18.8 Gy <5 cc (i.e., volume receiving 18.8 Gy dose is <5 cm^3^), including the NC plan. For the other OARs (ribs and heart), all investigated plans met the clinical dose threshold requirements [see Fig. 6(b) and Table 8].

### 3.D Marker visibility and trackability

In terms of quantifying trackability, Table 4 shows values for the percentage of the markers visualized at all VMAT gantry arc control points in the dynamic thorax phantom. For the NC plan, all three markers were visualized and trackable in 20% of the gantry control points; only two markers are trackable in 48.6%, only one trackable in 26.1%, and no markers trackable in 5.3% of the BEV and gantry control points.

**Table 4:** Marker visibility and trackability metric for the dynamic thorax phantom for all VMAT optimized plans (i.e., NC, SC, and HC) showing the percentage gantry control points whereby the fiducial markers were: all fully trackable, at least two trackable, and at least one trackable in BEV.

Constrained plans	% Control points with all three markers in BEV	% Control points with only two markers in BEV	% Control points with only one marker in BEV	% Control points with no marker in BEV	% Control points with marker 1 in BEV	% Control points with marker 2 in BEV	% Control points with marker 3 in BEV
SC1	48.2	37.8	14.0	NA	100	31.7	6.1
SC2	50.4	43.0	6.6	NA	34.3	100	8.7
SC3	92.9	7.1	‐	NA	7.0	0.1	100
SC12	61.3	38.7	NA	NA	100	100	61.3
SC13	90.9	9.1	NA	NA	100	90.9	100
SC23	97.0	3.0	NA	NA	97.0	100	100
HC	100.0	NA	NA	NA	100	100	100
**Reference/nonconstrained plan**	**% Control points with all three markers in BEV**	**% Control points with only two markers in BEV**	**% Control points with only one marker in BEV**	**% Control points with no marker in BEV**	**% Control points with marker 1 in BEV**	**% Control points with marker 2 in BEV**	**% Control points with marker 3 in BEV**
NC	20.0	48.6	26.1	5.3	51.6	48.6	23.1

BEV, beam's eye view; NC, nonconstrained; SC, soft constrained; HC, hard constrained; VMAT, volumetric modulated arc therapy.

**Fig. 9:**
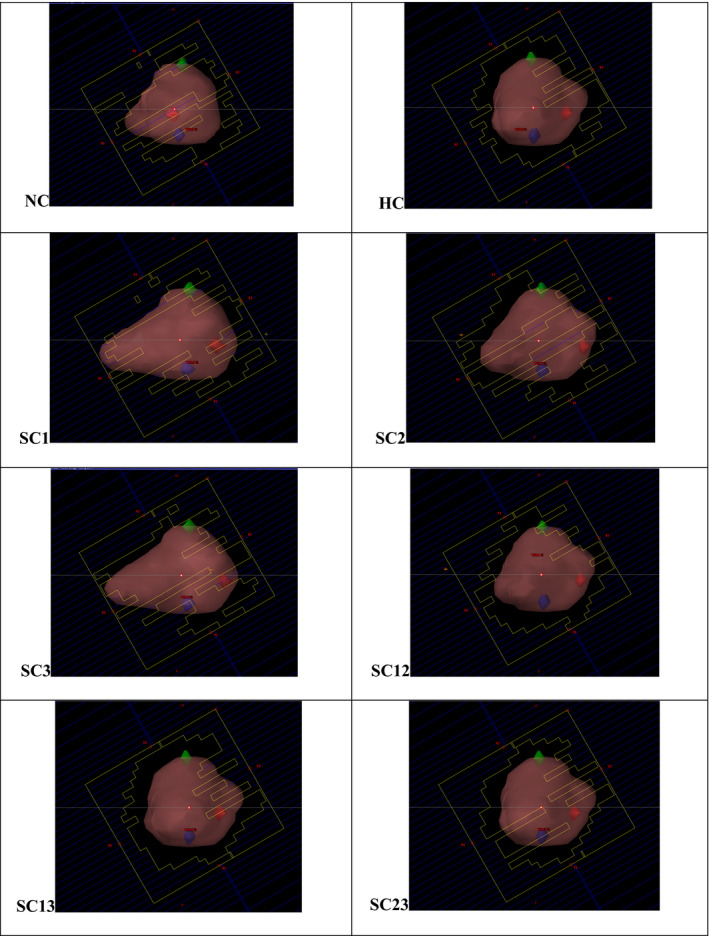
Example machine’s multileaf collimator (MLC) beams‐eye‐view (BEV) aperture at different gantry control points in nonconstrained (NC) and constrained (HC and SC) volumetric modulated arc therapy plans for the lung SBRT patient, with PTV and fiducial markers (1‐blue, 2‐red and 3‐green) either blocked and/or fully visualized by the MLCs.

Figure 9 shows an example MLC BEV aperture at different gantry arc control points for the NC, HC, and SC VMAT plans in the lung SBRT patient, respectively. As seen, the specific constrained markers are trackable in every control point of the gantry rotation. Similarly for trackability, Table 5 details the percentage of the markers visualized at all VMAT gantry arc control points in the lung SBRT patient. For the NC plan, all three markers were visualized and trackable in 15.22% of the total gantry control points; only two markers are trackable in 30.43%; only one trackable in 33.47% and no markers trackable in 20.87% of the BEV and gantry control points.

**Table 5:** Marker visibility and trackability metric for the lung SBRT patient for all VMAT optimized plans (i.e., NC, SC, and HC) showing the percentage gantry control points whereby the fiducial markers were: all fully trackable, at least two trackable, and at least one trackable in BEV.

Constrained plans	% Control points with all three markers in BEV	% Control points with only two markers in BEV	% Control points with only one marker in BEV	% Control points with no marker in BEV	% Control points with marker 1 in BEV	% Control points with marker 2 in BEV	% Control points with marker 3 in BEV
SC1	39.13	43.48	17.39	NA	100	60.87	60.87
SC2	45.65	48.26	6.09	NA	85.21	100	54.35
SC3	45.22	36.52	18.26	NA	73.48	53.48	100
SC12	57.39	42.61	NA	NA	100	100	57.39
SC13	67.80	32.20	NA	NA	100	67.8	100
SC23	88.26	11.74	NA	NA	88.26	100	100
HC	100.0	NA	NA	NA	100	100	100
**Reference/nonconstrained plan**	**% Control points with all three markers in BEV**	**% Control points with only two markers in BEV**	**% Control points with only one marker in BEV**	**% Control points with no marker in BEV**	**% Control points with marker 1 in BEV**	**% Control points with marker 2 in BEV**	**% Control points with marker 3 in BEV**
NC	15.22	30.43	33.47	20.87	41.30	42.61	51.73

BEV, beam's eye view; NC, nonconstrained; SC, soft constrained; HC, hard constrained; VMAT, volumetric modulated arc therapy; SBRT, stereotactic body radiation therapy.

**Fig. 10:**
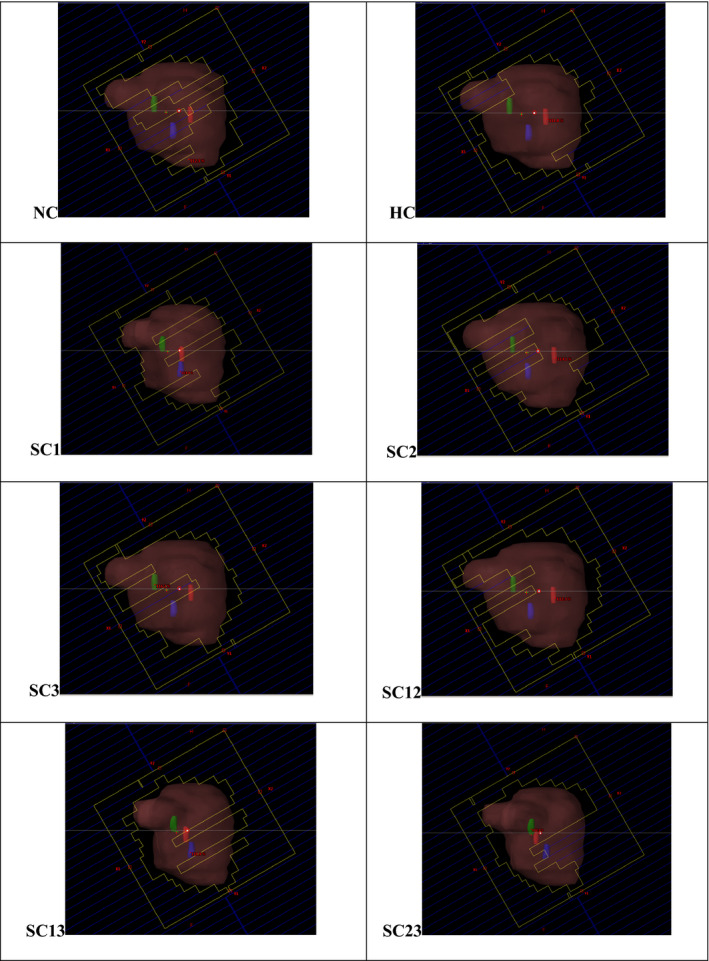
Example machine’s multileaf collimator (MLC) beams‐eye‐view aperture at different gantry control points in nonconstrained (NC) and constrained (HC and SC) volumetric modulated arc therapy plans for the prostate patient, with PTV and fiducial markers (1‐blue, 2‐red, and 3‐green) either blocked and/or fully visualized by the MLCs.

Figure 10 presents an example MLC BEV aperture at different gantry arc control points for the *MonArc*‐based NC, HC, and SC VMAT plans in the prostate patient, respectively. For the trackability metric, Table 6 shows values for the percentage of the markers trackable at all VMAT gantry arc control points in the sample prostate patient. For the NC plan, all three markers are trackable in 27.3% of the gantry control points; while only two markers are trackable in 30.2%, only one trackable in 20.4% and no markers trackable in 22.1% of the BEV gantry control points.

**Table 6:** Marker visibility and trackability metric for the prostate patient for all VMAT optimized plans (i.e., NC, SC, and HC) showing the percentage gantry control points in which fiducial markers were: all fully trackable, at least two trackable, and at least one trackable in BEV.

Constrained plans	% Control points with all three markers in BEV	% Control points with only two markers in BEV	% Control points with only one marker in BEV	% Control points with no marker in BEV	% Control points with marker 1 in BEV	% Control points with marker 2 in BEV	% Control points with marker 3 in BEV
SC1	81.0	15.6	3.4	NA	100	7.0	8.6
SC2	75.7	17.9	6.4	NA	3.4	100	14.5
SC3	79.9	18.4	1.7	NA	6.7	11.7	100
SC12	96.3	3.7	NA	NA	100	100	96.3
SC13	94.4	5.6	NA	NA	100	94.4	100
SC23	90.8	9.2	NA	NA	90.8	100	100
HC	100.0	NA	NA	NA	100	100	100
**Reference/nonconstrained plan**	**% Control points with all three markers in BEV**	**% Control points with only two markers in BEV**	**% Control points with only one marker in BEV**	**% Control points with no marker in BEV**	**% Control points with marker 1 in BEV**	**% Control points with marker 2 in BEV**	**% Control points with marker 3 in BEV**
NC	27.3	30.2	20.4	22.1	49.7	55.3	57.8

BEV, beam's eye view; NC, nonconstrained; SC, soft constrained; HC, hard constrained; VMAT, volumetric modulated arc therapy.

**Table 7:** Quality metrics for *MonArc*‐based optimized VMAT plans on the dynamic thorax phantom using: NC, HC, and SC. Thus, SC3, soft‐constrained plan for marker 3; SC12, soft‐constrained plan for markers 1 and 2, etc.

Metrics	SC1	SC2	SC3	SC12	SC13	SC23	HC	NC
PTV conformity index	1.72	1.71	1.69	1.64	1.62	1.66	1.55	1.52
PTV homogeneity index	1.18	1.18	1.19	1.16	1.18	1.18	1.17	1.19
PTV average index = (CI + HI)/2	1.45	1.44	1.44	1.40	1.40	1.42	1.36	1.35
PTV gradient measure (cm)	1.27	1.26	1.25	1.26	1.27	1.25	1.28	1.20
D_99%_ >95% of Rx dose (%)	97.83	97.40	97.70	97.83	98.13	97.30	97.83	97.87
PTV, *D_mean_* (%)	107.63	108.40	106.77	106.78	105.65	106.60	105.50	107.50
OAR spinal cord, *D_mean_* (%)	7.70	7.17	8.63	8.33	8.30	9.20	8.33	9.67
OAR vertebral body, *D_mean_* (%)	3.83	3.47	4.13	3.87	3.93	4.40	4.03	4.47
OAR lungs, *D_mean_* (%)	9.17	9.23	9.47	9.20	9.37	9.37	9.33	9.03
Beam output (MU)	1075	1075	972	1019	961	958	947	1098

PTV, planning target volume; OARs, organs at risk; NC, nonconstrained; SC, soft constrained; HC, hard constrained; VMAT, volumetric modulated arc therapy; CI, conformity index; HI, homogeneity index.

**Table 8:** Quality metrics for *MonArc*‐based optimized VMAT plans on the lung SBRT patient using: NC, HC, and SC. Thus, SC1, soft‐constrained plan for marker 1; SC23, soft‐constrained plan for markers 2 and 3, etc.

Metrics	SC1	SC2	SC3	SC12	SC13	SC23	HC	NC
PTV conformity index	1.36	1.29	1.25	1.33	1.28	1.30	1.30	1.25
PTV homogeneity index	1.09	1.11	1.11	1.09	1.10	1.11	1.10	1.10
PTV average index = (CI + HI)/2	1.23	1.20	1.18	1.21	1.19	1.21	1.20	1.17
PTV gradient measure (cm)	1.35	1.38	1.43	1.38	1.39	1.43	1.44	1.41
D_99%_ >95% of Rx dose (%)	97.0	97.0	96.8	96.5	96.8	97.2	96.4	96.9
PTV *D_mean_* (%)	110.1	109.8	109.3	109.5	108.3	109.3	109.3	109.8
OAR lungs, D30%* <*13 Gy (Gy)	2.19	2.20	2.14	2.21	2.12	2.06	2.10	2.10
OAR esophagus, V18* <*5 cc (cm^3^)	0	0	0	0	0	0	0	0
OAR ribs, V32 <1cc (cm^3^)	0.58	0.57	0.54	0.58	0.59	0.58	0.56	0.56
OAR heart, V28 <15 cc (cm^3^)	0.34	0.32	0.21	0.31	0.37	0.42	0.31	0.42
Beam output (MU)	1181	1138	1148	1151	1121	1142	1160	1181

PTV, planning target volume; OARs, organs at risk; NC, nonconstrained; SC, soft constrained; HC, hard constrained; VMAT, volumetric modulated arc therapy; SBRT, stereotactic body radiation therapy; CI, conformity index; HI, homogeneity index.

### 3.C Marker‐based constrained optimization on prostate patient

**Fig. 7:**
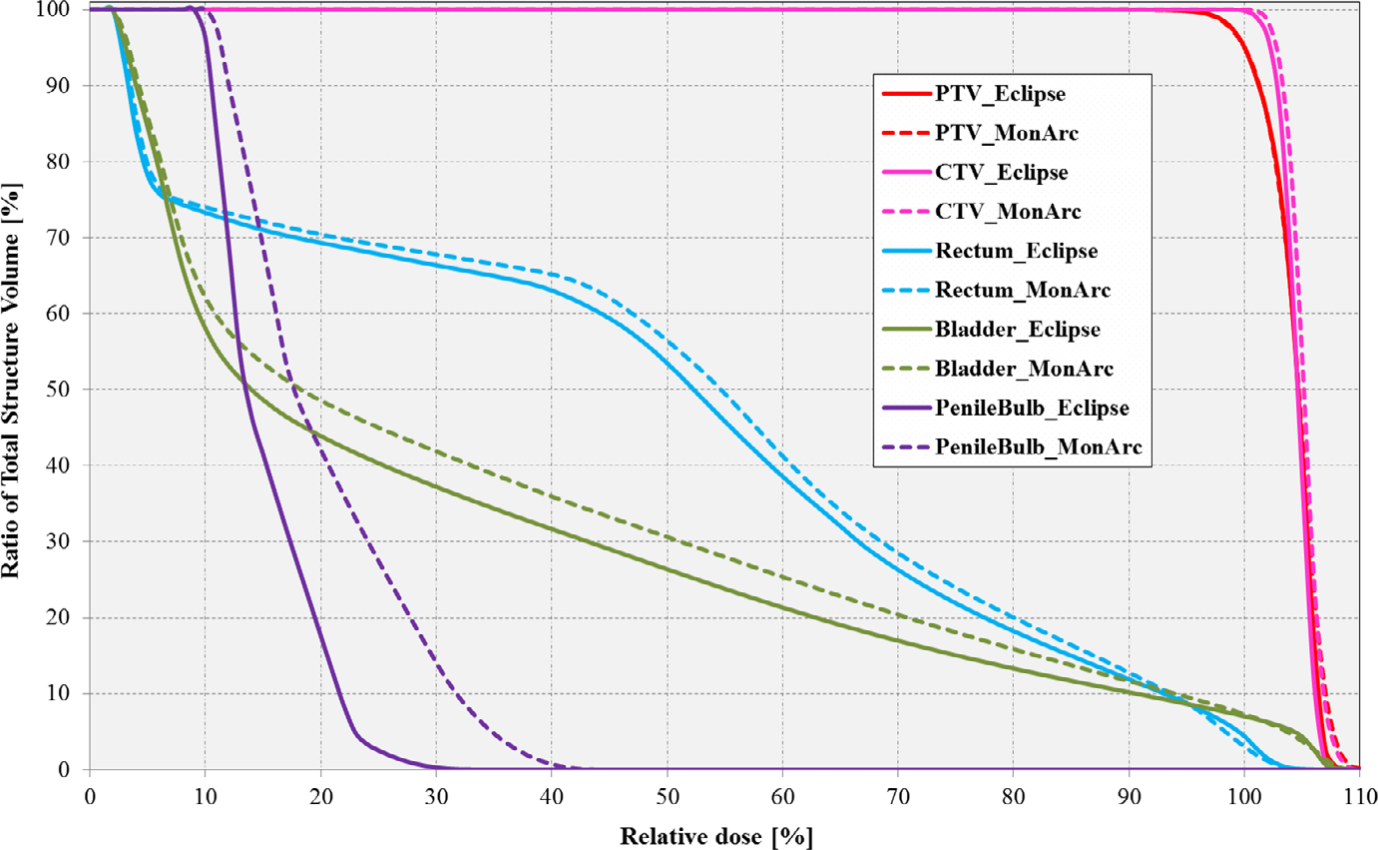
Dose–volume histogram comparison between Eclipse‐based and *MonArc*‐based volumetric modulated arc therapy plans for the target structures (CTV [pink]; PTV [red]) and organs at risks (Bladder [green]; Rectum [blue]; Penile Bulb [purple]) on the sample prostate patient. Both plans are nonconstrained on the markers.

Figure 7 shows the DVH comparison between the NC clinical and *MonArc*‐based patient prostate VMAT plans. As can be observed in this case, the *MonArc*‐based plan is nearly the same in terms of dose conformity to the target. Similarly, both plans passed the established site‐specific clinical dose tolerances for the target and OARs.

**Fig. 8:**
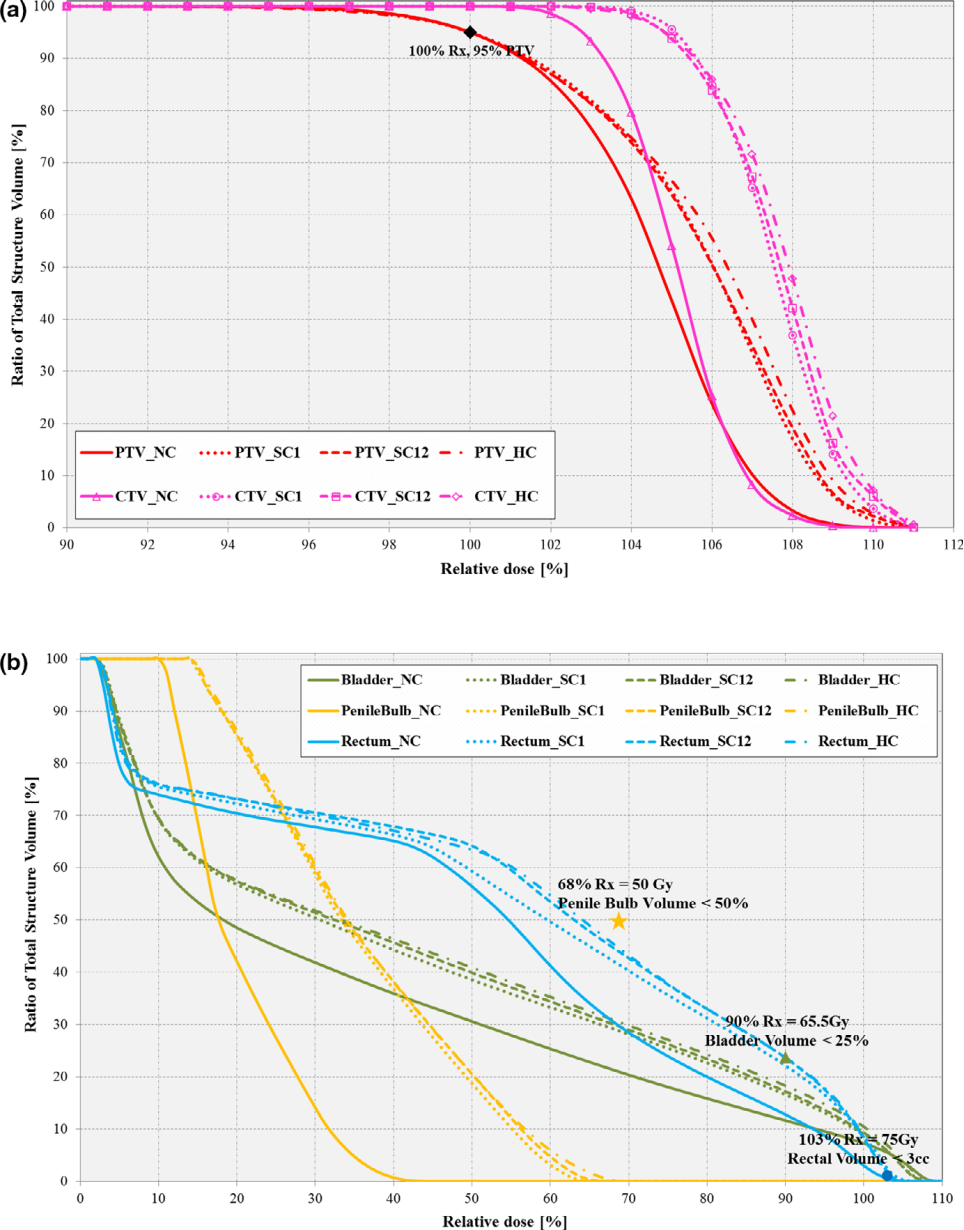
Dose–volume histogram comparison for (a) targets (CTV [pink] and PTV [red]) and (b) organs at risk (OARs) (Bladder [green], Penile Bulb [yellow] and Rectum [blue]) between *MonArc*‐based nonconstrained (NC), soft‐constrained (SC), and Hard‐constrained (HC) volumetric modulated arc therapy plans on sample prostate patient. Markers (circle, triangle, star) represent labeled OAR‐specific dose‐quality thresholds.

Figure 8(a) shows the target’s zoomed‐in DVH plots for the *MonArc*‐based NC, HC, and SC VMAT plans, respectively. As can be seen, the NC plan offered the best optimal dose coverage for the PTV target. For the SC plans, SC1 (i.e., Inferior marker) plan provided the most optimal dose–volume coverage while SC13 (i.e., Inferior + Superior marker) plan produced the least optimal dose coverage along with the HC plan. However, all the plans produced dosimetrically acceptable coverage and conformity for the PTV target as defined by local clinical standards. For the rectum OAR [see Fig. 8(b) and Table 9], all constrained plans met the clinical dose threshold V74 Gy <3 cc (i.e., rectal volume receiving 74 Gy dose is less than 3 cm^3^), including the NC plan safe for the SC13 plan which exceeded the value by 3%. For the bladder OAR, all plans were under the required value of 25% for bladder volume receiving 65 Gy. For the other OARs (bladder, head‐of‐femur, and penile bulb), all plans also met the clinical dose threshold requirements, except for the SC13 rectal coverage [see Fig. 8(b) and Table 9].

**Table 9:** Quality metrics for *MonArc*‐based optimized VMAT plans on the prostate patient using: NC, HC, and SC. Thus, SC2, soft‐constrained plan for marker 2, SC13, soft‐constrained plan for markers 1 and 3, etc.

Metrics	SC1	SC2	SC3	SC12	SC13	SC23	HC	NC
PTV conformity index	1.13	1.13	1.16	1.16	1.18	1.18	1.20	1.04
PTV homogeneity index	1.08	1.09	1.11	1.07	1.06	1.07	1.06	1.08
PTV average index = (CI + HI)/2	1.11	1.11	1.13	1.12	1.12	1.13	1.13	1.06
PTV gradient measure (cm)	1.89	1.92	1.97	1.94	1.94	1.91	2.03	1.79
D_99%_ >95% of Rx dose (%)	98.0	97.5	97.8	97.9	97.5	97.5	97.7	97.5
PTV *D_mean_* (%)	104.7	104.7	104.9	104.8	105.3	105.0	105.4	104.0
OAR bladder, V65 *<*25% Rx (%)	14.26	14.80	15.13	15.27	14.99	15.66	15.75	11.99
OAR rectum, V74 *<*3 cc (cm^3^)	1.88	2.06	2.45	2.42	2.91	3.10	2.31	1.35
OAR penile bulb, *D_mean_* <50 Gy (Gy)	16.09	15.91	15.83	16.19	15.78	16.36	16.33	13.67
Beam output (MU)	499.6	491.3	502.9	477.7	478.5	480.9	470.2	613.4

PTV, planning target volume; OARs, organs at risk; NC, nonconstrained; SC, soft constrained; HC, hard constrained; VMAT, volumetric modulated arc therapy; CI, conformity index; HI, homogeneity index.

### 3.D Comparison metrics

Table 7 shows the quantitative dose quality metrics (i.e., CI, HI, and GM) for all the lung VMAT plans on the dynamic thorax phantom for the NC, HC, and SC plans, respectively. The lowest CI value of 1.52 belongs to the NC plan, followed by the HC plan with 1.55 CI. This implies that the NC plan produced the best conformal plan. Using the GM, the NC plan produced the best dose falloff slope with a 1.20 GM value, followed by SC3 (1.25 GM) and SC23 (1.25 GM) plans while the HC plan (1.28 GM) produced the worst gradient value. Similar results were obtained using the HI values. The beam output monitor units (MU) were highest with the NC plan, whereby the NC plan produced the highest output of 1098 MU, while the HC plan produced the lowest output of 947 MU.

Table 8 shows the quantitative dose quality metrics (i.e., CI, HI, and GM) for all the lung VMAT plans on the lung SBRT patient for NC, HC, and SC plans, respectively. The lowest CI value belongs to the NC plan (CI = 1.24), followed by the SC3 plan (CI = 1.25) and SC13 plan (CI = 1.28). This implies that the NC plan produced the best conformal plan. Using the GM, however, the SC1 plan produced the best dose falloff slope with a 1.35 GM value, followed by NC (GM = 1.38) and SC12 (GM = 1.38) plans while the HC plan (GM = 1.44) produced the worst gradient value. Similar results were obtained using the HI values. Using the average of the index values, the NC plan produced the best conformal and homogenous plan. The beam output MU were highest with the NC plan, whereby the NC plan produced the highest output of 1181 MU, while the SC13 plan produced the lowest output of 1121 MU.

Similarly, Table 9 details the quantitative dose quality metrics for the prostate VMAT plan on the prostate patient with the visibility constraints: NC, HC, and SC. The lowest CI value of 1.04 belongs to the NC plan, followed by SC2 and SC3 plans (CI = 1.13) while HC plan had the worst CI value of 1.20. Similarly, the GM values were also lowest with the NC plan, while the HI values were lowest with the SC12 plan. However, using the average index, the NC plan still showed the lowest value, followed by the SC2 plan and the lowest with the HC plan. This shows that the NC plan produced the most optimal conformity, dose falloff slope, and homogeneity. In terms of the treatment beam output in MU, the HC plan produced the lowest beam output value of 470 MU, while the NC plan produced the highest beam output at 613 MU.

## 4 Discussion

The best dosimetric target coverage for the PTV in the dynamic thorax phantom — as indicated by D_99%_, CI, HI, and GM — was produced by the NC plan, followed by the HC and SC plans (Table 7). Since no singular quantitative metric is adequate to describe the best plan, an average of HI and CI will be most appropriate. Using this average, the NC plan still provided the best quantitative metric score. Hence, it can be deduced that the NC plan produced the most dosimetrically optimal plan for the thorax phantom. This is expected as the NC plan has no marker visibility constraints in its implementation.

The difference in the metrics between HC and SC plans were not significant. However, we did not anticipate the HC plan to produce better or similar results compared to SC plan as the HC plans are constrained such that the markers are fully visualized at all gantry angles in the beam’s eye view, thereby forcing larger MLC fields. Furthermore, the beam output MU were highest with the NC plan. This is expected as there are no fiducial marker visibility constraints in the optimization process for NC plans, so the MLC aperture is more highly modulated to achieve the dose objectives. Alternatively, both the SC13 and HC plans are constrained to see at least two markers, making the beam less modulated as the MLCs are required to be open to visualize two markers.

OAR dosimetry results for the thorax phantom (see Fig. 4) do not clearly follow the pattern observed for targets, with the SC_I_ plan (SC_I_ = SC1, SC2, or SC3) outperforming the HC and NC plans. For the spinal cord, the mean dose was between 8.3% and 9.7% across all plans, while for the vertebrae the mean dose was between 3.5% and 4.5%. For the lung critical structure, the mean dose across all investigated plans was between 9.0% and 9.5%, with no noticeable over or under‐dosage for all investigated plans. The discrepancy in target and OAR results for the phantom may be due to the very simple geometry of the phantom, and position of the simulated fiducial markers. However, these preliminary results demonstrate that visibility constraints may be included in plan optimization.

In terms of the trackability metric for the phantom (Table 4), tracking of all three markers is feasible in at least 20% of the gantry rotation in the reference NC lung VMAT plan. For the SC plans in the thorax phantom, all three markers are trackable between 48% and 97% of the available gantry arc rotation depending on marker location, and 100% for HC plans. The SC plans show increased coverage as the constrained markers increased from one specific marker (SC_I_ = SC1, SC2, or SC3) to two specific markers (SC_II_ = SC12, SC13, or SC23). This shows that as the plans are constrained to track more than one marker, the MLCs open to accommodate the specified markers such that more or all inserted/implanted markers are in the BEV such that aperture fields are inversely proportional to beam modulation, resulting in less beam output (MU).

For the VMAT lung SBRT patient dataset, the best dosimetric target coverage [Fig. 6(a)] for the PTV as indicated by the quality metrics (Table 8) was produced by the SC13 and NC plans. The worst coverage was provided by the SC23 and HC plans. However, there was no significant disparity in target coverage among all plans. OAR dosimetry results for the lung patient [Fig. 6(b)] shows that all plans consistently met the clinical dose objectives. However, some SC_I_ plans (e.g., SC3 plan for the Superior marker) outperformed the NC plan, exposing the heart OAR to up to 50% less dose relative to the reference NC plan. In terms of the beam output, the NC plan also produced the highest output as measured in MU, as was observed in the phantom.

For the example VMAT lung SBRT patient plan, trackability of all the markers is feasible in at least 15.22% of the gantry rotation in the reference NC lung VMAT plan, while none of the fiducial would be available for tracking in at least 20.87% (Table 5) of the treatment delivery time. However, for the SC plans in the lung patient, all three markers were trackable between 39% and 88% of the available gantry arc rotation. The SC plans also exhibited increased coverage as the constrained markers visualized improved from one (SC_I_ = SC1, SC2, or SC3) to two specific markers (SC_II_ = SC12, SC13, or SC23). This also shows SC plans approach the HC plan as more markers are included in the optimization and the MLCs open to accommodate the specified markers.

For the VMAT prostate patient dataset, the best dosimetric target coverage [Fig. 8(a)] for the PTV as indicated by the quality metrics (Table 9) was produced by the NC plan. All other constrained plans (SC and HC) had similar metrics with no significant disparity in target coverage. OAR dosimetry results for the prostate patient [Fig. 8(b)] shows that all plans consistently met the clinical dose objectives, except for the rectal threshold for SC23 plan. In general, the bladder, rectal, and penile bulb dose objectives were met by all the plans. Nevertheless, some SC plans slightly outperform the HC plans, but they all expose the OARs to up to 4% more dose relative to the reference NC plan.

Similarly, trackability of all the markers (Table 6) is feasible in at least 27% of the gantry rotation, while none of the fiducial would be available for tracking in at least 22% of the treatment delivery time. However, in the SC prostate plans, all three markers were trackable between 75% and 96% of the available gantry arc rotation. The SC plans also exhibited increased coverage as the constrained markers visualized improved from one (SC_I_) to two specific markers (SC_II_). This also shows SC plans approach the HC plan as more markers are included in the optimization and the MLCs open to accommodate the specified markers.

The approach presented by Azcona *et al.* ^11^ to predict the marker positions for prostate patients, where markers are unavailable or blocked by the MLCs, can be useful in addressing some of the shortcomings for clinical VMAT. However, it requires image filtering and template selection that needs to be small enough to allow real‐time processing. The authors point out that the technique will also suffer from sparse data and long successive control points where markers are unavailable. As detailed in the literature, the motion of the tumor needs to be accounted for during the imaging, planning, and treatment delivery stage such that a temporal component can be included in 4D radiotherapy.^20^ Methods to obtain 4D radiotherapy can be either via moving a robotic gantry to adjust the radiation beam position with the tumor position, or alternatively by dynamic MLC motion adjustments, or by moving the patient via couch motion.^20^, ^21^ However, to perform real‐time tumor tracking robustly, the visibility of fiducial markers in the small and irregularly shaped fields found in arc radiotherapy (such as 4D‐VMAT) needs to be assured.

A limitation of our work is that we have not studied the effects of the residual motion between the tumor and the healthy tissues when respiration is accounted for (i.e., this feasibility study is not performed in 4D). However, a 4D‐VMAT implementation introduced by Chin et al.,^14^, ^15^ could be modified to account for the marker motion relative to the motion of the OARs. A future goal of our research includes such an implementation. Furthermore, since only feasibility is demonstrated in this study on limited patient data, we intend to apply our method to more clinical datasets with varying characteristics, to understand the effects of the algorithm on more patient datasets with different tumor/disease sites. Such work is ongoing and will be disseminated in another publication in the near future.

We have shown the feasibility of adding marker visibility parameters into the optimization objective function, successfully combining them together with standard dose objectives to obtain clinically acceptable plans. This current work can be extended to investigate the marker‐based constrained optimization for 4D‐VMAT. There is also interest in real‐time tumor tracking without markers (i.e., markerless tracking methods), and those strategies also have visibility requirements of the target. Our proposed approach could also be similarly applicable to markerless tracking methods when incorporated into the optimization.

## 5 Conclusion

The results presented in this study show the preliminary evaluation of including fiducial marker visibility objectives together with dose objectives in the optimization of VMAT treatment plans, and their success achieving both types of objectives. We have been able to test it on a dynamic thorax phantom as well as on a lung and prostate patient dataset. Our results show that plans can be developed that satisfy visibility requirements for trackability of fiducial markers, while still achieving dosimetric objectives of the target and OARs. Using SCs provide acceptable dosimetric tolerances for both the target and OARs for both the lung and prostate patient dataset. However, OAR doses may be increased or reduced depending on the location and number of constrained markers in the MLC BEV. In conclusion, our results show that the tumor visibility and trackability may be improved by adding appropriate fiducial marker constraints in the optimization process, while still preserving dosimetric plan quality.

## Conflict of Interests

The authors have no conflicts of interest to disclose.
